# Helping behaviour of volunteers in providing post-disaster psychosocial assistance

**DOI:** 10.4102/jamba.v17i1.1792

**Published:** 2025-05-26

**Authors:** Nevi K. Arianti, Muhammad Baiquni, Koentjoro Soeparno, Arifin Nur Afni I.

**Affiliations:** 1 Faculty of Psychology, Universitas Gadjah Mada, Yogyakarta, Indonesia; 2 Faculty of Geography, Universitas Gadjah Mada, Yogyakarta, Indonesia

**Keywords:** post-disaster, helping behavior, volunteer, psychosocial, local wisdom

## Abstract

This study explores the ways that volunteers help in overcoming the negative impacts of disasters. The direction of this research is the production of guiding data for roadmaps for strengthening the capacity of volunteers as the spearhead of disaster risk reduction. The purpose of the study was to find out how the volunteers’ helping behaviour provides post-disaster psychosocial support. Two kinds of studies (multimethod) were conducted to answer the research questions. Study 1 was conducted on three volunteer participants, using the phenomenological method. Analysis of research data using interpretative phenomenological analysis. Study 2 was conducted on 69 volunteer respondents, using a survey method with open-ended questions to determine respondents’ perceptions of the meaning of helping and the meaning of psychosocial support. The results of data integration found five themes in helping behaviour which included the values of volunteers and their goals, motivation and attitudes in helping, cooperation with other parties, as well as special themes/other findings in the form of mottos owned by volunteers.

**Contribution:**

The meaning of helping volunteers, which is a form of caring, satisfaction, self-meaningfulness, self-spirituality, behaviour that aims to lighten the burden on others, restore other people’s conditions to be better, and provide assistance. The meaning of psychosocial support includes seven themes, namely: material, non-material, relieving the burden, dealing with certain circumstances or objects, relating to the subject or person, recovery and help or support.

## Introduction

This study examines the helping behaviour of volunteers in providing psychosocial assistance or support in the post-disaster period. What is meant by volunteer helping behaviour in this study is any psychosocial assistance behaviour carried out by volunteers to disaster survivors in the post-disaster period (Henley 2005). The post-disaster period is the time after the disaster emergency/emergency period, namely the recovery phase (Olshansky & Johnson 2014). Helping behaviour is something that looks simple, but when assistance or help is not targeted, for example, given to people who do not need it, or done inappropriately, it can add to existing problems and even threaten the survival of beneficiaries and even the community together (Grusec et al. 2002). One of the impacts of problems that can arise is horizontal conflicts between residents in the community during disasters and post-disasters as well as vertical conflicts, namely the community with managers/structures (Grusec et al. 2002).

Kachukhova et al. (2021) suggest that helping behaviour is categorised into several domains, including instrumental helping, comforting others, and sharing resources. Wilhem and Bekkers (2010) mentioned that helping behaviour is studied within various frameworks, including social evolutionary theory, ecological settings, game theory, and social cognitive requirements. These approaches differ in their focus on the mechanisms and conditions that support helping behaviour, such as direct and indirect benefits, interdependence, and strategies to protect oneself from cheaters. Chou and Stauffer (2016) believe that helping behaviour can be classified based on the request of the beneficiary and the helpfulness of the helper. The three forms identified are unsolicited proactive helping, unsolicited reactive helping, and solicited reactive helping. Each type is associated with different motives and requires different levels of co-operation and sacrifice.

Hogg and Vaughan (2002) suggested that helping behaviour is a behaviour that provides more benefits to other individuals than to oneself. Baron and Byrne (2005) then emphasised that helping behaviour is an action that provides direct benefits to other individuals, without providing benefits to the individual who provides help. Helping behaviour is a behaviour that arises from trust and cooperation. Schein (2009) suggests that helping behaviour is a complex thing that can occur in all situations. Even though the help provided might be helpful, many might not gain from it at all. Humans as social creatures certainly need help in everyday life, but often individuals have difficulty or even fail to provide help. Furthermore, Schein (2009) stated that helping is commonly found in everyday life. Helping is the act of assisting another individual’s task so that it becomes easier and can be completed. Helping behaviour in individuals can arise spontaneously when individuals are aware that other individuals need help, even without being asked (Laguna et al. 2020).

There are several principles regarding aid, namely: assistance is given to the right target; the type and amount of assistance do not cause conflict and assistance should not create dependence but rather empower the community or beneficiaries (interview with Kharismawan and Krisdhanta, consultants and psychosocial activists, September 2021). When helpers provide assistance, the principle of assistance is important to apply in disasters so that any assistance delivered is part of the social or psychosocial support process. In disaster situations, whether natural hazards, social disasters or the current COVID-19 pandemic, assistance in the form of social or psychosocial support is needed in addition to material assistance. Psychosocial assistance is not only limited to clinical counselling or work therapy, for example, but also the entire process of channelling assistance to beneficiaries (Rodriquez et al. 2018).

Helping or what can also be called helping behaviour, is an action that aims to prosper others by being driven by selfish and altruistic motives (Marjanovic, Struthers & Greenglass 2012). Amato (1990) distinguishes the forms of helping behaviour into two, namely spontaneous helping and planned helping. Planned helping itself is further divided into two forms. Firstly, formal planned helping, which is helping behaviour aimed at helping individuals or groups through agencies or organisations. Secondly, informal planned helping is helping behaviour aimed at people who are already known and have closeness, such as friends or family members. Then, spontaneous helping is helping behaviour aimed at strangers we do not know. This behaviour occurs suddenly or spontaneously and is not planned in advance.

Volunteer helping behaviour is classified as formal planned behaviour. The results of Utomo and Minza’s (2018) research show that there is a change in the form of helping behaviour from spontaneous helping behaviour, or called spontaneous helping, to planned helping behaviour or planned helping in the three informants who became spontaneous volunteers. The impetus that gave rise to spontaneous helping behaviour in him came from the conditions seen and heard by the informants which then gave rise to feelings of empathy and curiosity about the location of natural hazards. Kassin, Fein and Markus (2014) suggest that there are three aspects of helping behaviour, namely: reward, empathy, altruism and egoism. (1) Reward is the reward that individuals receive when they help. One of the reasons individuals help is to obtain psychological and material rewards. Individuals often feel happy when helping other individuals. (2) Empathy is the feeling of understanding an individual’s perspective either directly or indirectly and feeling sympathy or love from that individual. (3) Altruism and egoism are two things that cause individuals to perform helping behaviour. Individuals who help based on altruism have a desire to improve the welfare of other individuals and do not think about the rewards they will receive. Individuals can also engage in helping behaviour due to egoism, which is the desire to improve their own well-being. Individuals perform helping behaviour to increase positive feelings within themselves.

The Global Platform Disaster Risk Reduction (Information GPDRR 2022, 2021) emphasised the importance of disaster risk prevention and reduction if a sustainable future is to be achieved, demonstrates the importance of international solidarity and cooperation, and discusses ways to address the underlying drivers of risk both locally and globally. The 2022 Global Platform not only speaks in terms of discourse but also provides an opportunity to share best practices and recommendations for policy makers (komdigi.go.id, 2022). Governmental and non-governmental organisations work together to help disaster victims from the pre-disaster, disaster and post-disaster stages. Volunteers are formed and join organisations or work independently. Some volunteers come from an open recruitment process, while others go through a closed recruitment process where volunteers are invited/involved by the volunteer network (Utomo & Minza, 2018). Volunteers have an important role to play in the “engine” and dynamics of the aid distribution process to disaster victims. Various problems and solutions when helping, are experienced by volunteers in the field. From in-depth interviews with several staff policymakers at Badan National Penanggulangan Bencana (BNPB, 2021), information was obtained on how challenging the design, process and implementation of aid distribution for disaster-affected residents is. Volunteers who work to help disaster-affected people together with government and non-government organisations and community members certainly have valuable experience. Koentjoro & Andayani (2007) say that the helping behavior of disaster volunteers needs to be based on four Community Development approaches, namely: 1) Awareness that helping is not an easy job; 2) Helping must optimise the role of the person being helped; 3) Helping must empower the person being helped; and 4). Helping must prosper the person being helped, instead of causing dependence on disaster survivors. Therefore, volunteers need to pay attention to several principles regarding assistance, namely: assistance is given to the right target; the type and amount of assistance does not cause conflict, and assistance must not create dependence, but rather empower the community or beneficiaries.

One type of assistance provided to communities experiencing disasters is psychosocial assistance. The Ministry of Women’s Empowerment and Child Protection (Kemenpppa) uses the term ‘support’ for the psychosocial assistance provided. Chaplin (2006) suggests that psychosocial is a psychological aspect that explains social relations that include various aspects of psychology. Baron and Byrne (2005) suggest that psychosocial knowledge seeks to understand the causes of individual behaviour and thinking in the context of social situations. According to Kemenpppa (data), psychosocial is a dynamic relationship between the psychological and social aspects of a person. Psychosocial support is any form of support from local or external parties that aims to maintain or promote psychosocial well-being and/or prevent or overcome mental disorders. Thus it can be said that psychosocial support is a form of assistance provided to individuals who experience psychological disorders due to disasters. The form of psychosocial support is carried out continuously and mutually influences the psychological and social aspects of the individual’s environment. Smet (1994) suggests that there are four dimensions of psychosocial support, namely: (1) Emotional support in the form of expressions of feelings of care, empathy and concern for other individuals; (2) Appreciative support in the form of positive expressions as a form of respect for other individuals; (3) Instrumental support in the form of direct assistance in the form of materials; and (4) Informative support in the form of providing advice, suggestions or feedback to individuals.

Volunteers, especially those on the front lines of disaster-prone areas, are the spearheads of the success of disaster management programmes. In order for the purpose of providing assistance to be achieved optimally, it is necessary to study how volunteer helping behaviour is. It is hoped that through this research, an appropriate form of psychosocial support for post-disaster victims can be obtained. Thus, the question posed in this study is how the helping behaviour of volunteers provides post-disaster psychosocial support. This question will be revealed by two studies, namely: (1) What is the meaning of helping disaster volunteers? (2) What is the perception of helping and post-disaster psychosocial support?

## Research methods and design

### Study 1

#### Participants

Research participants were determined using a purposive sampling technique by selecting subjects who fit the criteria that had been determined as follows: (1) Currently still active as a disaster volunteer. These participant criteria were chosen to understand the current condition of volunteers and not only past experiences. (2) Willing to be a research participant through informed consent. There was one participant who met these criteria: 45 years old, male, living in Yogyakarta and involved in various freelance volunteer groups and short-term activities from non-governmental organisations. The participant has experience as a volunteer in several types of disasters including volcanoes, floods, landslides, as well as earthquakes, tsunamis and the COVID-19 pandemic disaster (Table 1).

**TABLE 1:** Demographics of participants in study 1.

Participants	Age (years)	Gender	Daily activities	Residency
1	45	Male	Staff of Baguna Volunteer Community	Kulon Progo Regency, D.I. Yogyakarta
2	38	Male	Tagana Volunteer, Entrepreneur	Bantul Regency, D.I. Yogyakarta
3	48	Female	Goat Farmers and Breeders	Sleman Regency, D.I. Yogyakarta

D.I., Daerah Istimewa.

The approach used in this research is a phenomenological qualitative approach that focuses on the personal experiences of participants in the first person (Packer 2017). The phenomenological approach focuses on the awareness of human experience to find meaning, which is the main concept of phenomenology (Kahija, 2017; Hayes, 1997). The data collection method uses semi-structured interviews, namely by using an interview guide that has been made before the interview session takes place. Examples of item questions for study 1 are: (1) How do you interpret helping behaviour? (2) What do you feel when helping in disasters? (3) How do you respond to self-limitation in helping?

#### Data analysis

The research data were analysed using interpretative phenomenological analysis (IPA) to extract the meanings that emerged according to the participants’ context. The IPA technique was chosen because it is able to explore the important life experiences of individuals in natural settings (Smith, Flower & Larkin 2009). Data were tested for validity using source triangulation by interviewing participants’ significant others. In this study, the significant others interviewed were the volunteer’s wife and fellow disaster volunteers.

### Study 2

#### Participants

This study uses explorative qualitative according to Hickey and Kipping (1996) which aims to find themes related to life experience and participants’ perceptions of the meaning of helping and psychosocial support assistance. Research participants were determined using a purposive sampling technique, by selecting subjects who fit the inclusion criteria: 18–70 years old and natural hazard volunteers. There were 113 respondents who filled out the questionnaire, but 44 people did not qualify to continue because they did not fit the criteria as disaster volunteers. The participants whose data were processed totalled 69 people, consisting of 24 female participants (34.8%) and 45 male participants (65.2%) with a mean age of 41.6 years (s.d. = 11.6, see Table 2). Participants were spread from several regions both in villages and in big cities including Java (Kulon Progo, Yogyakarta, Kediri, Surabaya), Sumatra (Medan), Sulawesi (Makassar, Palu, Sigi, Donggala) and NTT (Kupang). The survey questions have been validated through multiple reviews by disaster experts, disaster volunteers and supervisors to ensure the reliability of the research instrument. Data collection was carried out using a survey method using open questions through the Google Form application. The question items are as follows: (1) What do you think helping means? (2) What do you think psychosocial assistance means?

**TABLE 2:** Demographics of participants in study 2 (*N* = 69).

Demographic characteristics	*n*	*f*(%)
**Gender**		
Female	24	34.8
Male	45	65.2
**Age category**		
Lowest age	21	-
Highest age	67	-
Mean age	41.6	-

#### Data analysis

The data in this study are participants’ responses to open-ended questions which are processed through MAXQDA 2020 for systematic coding and data management. This research applied a reflective thematic analytic process to understand participants’ perceptions and experiences (Braun & Clark 2012) as disaster volunteers. This method was also used to identify patterns in the themes reported by the research participants. The first, second and assistant authors familiarised themselves with the data set by repeatedly reading and making marks and notes. The first researcher, assisted by the second researcher and assistant, conducted independent initial codes, then continued with discussion and agreed on consensus between different codes. The meeting continued to further solidify the findings of themes that were in line with the consensus until finally there was agreement. The purpose of this process is to maintain the quality of the findings. The first and second researchers then reviewed the findings of themes and codes and then reached consensus on the final themes and sub-themes and their definitions.

### Ethical considerations

Ethical clearance to conduct this study was obtained from the Universitas Gadjah Mada Research Ethics Committee of the Faculty of Psychology (No. 12145/UN1/FPSi.1.3/SD/ PT.01.04/2023).

## Results

### Study 1

The findings obtained in Study 1 are themes that describe the experience of helping disaster volunteers. The main theme obtained is the meaning of helping behaviour or actions for volunteers and important values for the sustainability of their helping behaviour in the midst of difficult challenges and situations (Table 3). On the other hand, the superordinate themes found related to participants’ cognitive functions, feelings and behaviours and values that influence the experience of participants willing to survive to continue helping as volunteers.

**TABLE 3:** Main and superordinate themes.

Main themes	Superordinate themes
The meaning of helping	Caring, Satisfaction, Self-spirituality, Self-meaningfulness
Important values for the sustainability of their helping behaviour	Willingness, Openness to Learn, Willingness to Sacrifice or ‘Ngawu’ and Flexibility

The research also found several themes that are mottos for volunteers. The research finding in the form of a special theme is the volunteer motto. The motto is a special theme because it turns out that there are special findings in helping behaviour, where the motto of volunteers is considered by participants as an influential factor in the sustainability of disaster volunteer helping behaviour. There are four kinds of disaster volunteer motto themes (Table 6):

- Come-do-go home-Forget. Participants said that one of the efforts to maintain personal well-being as a volunteer is to be able to sincerely and quickly ‘move on’ and letting go of what is done.
- Menang Cacak Kalah Cacak, where disaster volunteers need to realise that the situation is uncertain and there is no certainty, so it is necessary to keep doing something for the good rather than not trying at all on the existing problems. Participant 1 learnt to take action and not be afraid of being wrong when volunteering. Participant 2 stated that she was more willing to be blamed for her decision, rather than not doing anything to help.
- Family is something that should not be defeated even in the name of helping. Volunteers are individuals who have left a lot of family in terms of time, thoughts and materials. Therefore, if there is an important family need, participants have the confidence for themselves and their team to just leave their volunteer work and focus on taking care of their family. Volunteer work can be delegated or completed by other colleagues, rather than the family being destroyed when it is not completed immediately. For example, when a volunteer is told by his wife that his child is sick, then another volunteer will remind the volunteer to go home earlier than the agreed time.
- Do not get carried away by posting counterproductive social media statuses. For example, when one participant is having family problems or work conflicts, other volunteers remind each other not to easily make statuses without enough emotional control. This can lead to an increased risk of more problems such as misunderstandings between disaster volunteers.

This research also found four superordinate themes that show the meaning of helping disaster volunteers (Table 3):

- **The presence of caring.** Participant revealed that caring is an important part of the meaningfulness of helping in disaster situations.
- **Spirituality of Self.** There is a push of self-emotion that becomes a force for the emergence of helping behaviour. The most dominant emotion that arises is pleasure, when it is happy, it becomes willing and willing, it becomes a strong intention to go and do the task of helping. There is a difference in the third participant, namely because of her position as a woman, the pleasure of helping is still filtered by the conflict of domestic priorities. That is, for example, how to see the condition of the children at home when she leaves for a volunteer meeting.
- **Self-meaningfulness.** Participants said that the reason for their helping behaviour is because when they help, they find the meaning of their life, the meaning of their presence in the community where they are. Self-existence and self-acceptance are important for all three participants.
- **Satisfaction.** The participant expressed her limited education compared to other volunteers in general. However, he was able to be confident and still proud to be able to dialogue and share the burden with volunteers from the campus, such as professors, doctors, clinicians and other highly educated social workers. The second participant said that her life became a blessing and felt happier when she was ready to accept rapid changes in the field while working. Meanwhile, the third participant was able to learn more flexibly when accepting the conditions as a female volunteer who was different from men. She no longer protests when there is different treatment because she realises differences as opportunities to complement each other.

### Study 1

This study found five themes identified as helping meanings and seven themes identified as psychosocial meanings that participants reported in the open-ended question. The sub-themes of ‘Obligation’, ‘Awareness’, ‘Acts of Love’ and ‘Humanism’ provide information on the theme of ‘Values held by volunteers’ in performing helping behaviour. The sub-themes of ‘Relief’ and ‘Giving help’ show the theme of ‘Purpose in helping’. The sub-themes ‘Making yourself happy’ and ‘Impulse’ show the theme ‘Motivation of helping behaviour’. While the sub-themes ‘Sincerity or Willingness’ and ‘Empathy’ show the theme ‘Attitudes that need to be possessed when helping’, the sub-theme ‘Connecting with others’ shows the theme ‘Cooperation with other parties’ in helping behaviour. The themes synthesised in research study 1 can be further seen in Table 4.

**TABLE 4:** Theme findings meaning of helping.

No	Theme	Sub-theme
1	Values held by volunteers	Obligation Awareness Acts of Love Humanism
2	Purpose in helping	Relief Giving help
3	Motivation of helping behaviour	Making yourself happy Impulse
4	Attitudes that need to be possessed when helping	Sincerity or Willingness Empathy
5	Cooperation with other parties	Connecting with others

No, number.

#### Theme 1: Values held by volunteers

This theme summarises how disaster volunteers in carrying out helping activities are based on intrinsic values, namely considering helping as an obligation; there is awareness from oneself to want to move to do something good for others. Participants also considered helping behaviour as an act of love and contained humanism values that respect human values. The disaster that occurred encouraged the emergence of the deepest values in volunteers in the form of helping behaviour, as a reflection of the embedded values so far in their life experience. A volunteer expressed her reflection when interpreting the meaning of helping, ‘An effort given consciously to others as an act of love and appreciation for the lives of others’ (RESP-30, 47 years old, female).

#### Theme 2: Purpose in helping

Disaster volunteers in deciding on helping behaviour are certainly based on goals. The purpose of the helping behaviour is aimed at the subject being helped, where the subjects being helped are disaster survivors who have a burden that needs to be relieved. Disaster survivors really need help both psychologically and physically. ‘Helping to ease someone’s burden both physically and mentally’ (RESP-23, 24 years old, female). The sensitivity of helpers, namely volunteers, to see what the needs of disaster survivors are is very important because the purpose of helping is to help lighten the burden and provide assistance according to needs, not assistance that does not meet the needs of disaster survivors. This was expressed by a volunteer, ‘To give and ease the burden of others’ (RESP-11, 23 years old, female). Helping is not easy because it requires the ability to assess, design, decide and execute from aid design to actual help. This was expressed by a volunteer, ‘Giving something material [*or*] non-material to others according to what is needed’ (RESP-17, 44 years old, male).

#### Theme 3: Motivation of helping behaviour

Helping is a behaviour that is certainly based on certain motivations. Participants’ motivation to help is divided between internal motivation and external motivation. Internal motivation is seen from the responses included in the theme ‘Make yourself happy’, which means that there is a happy emotion that is intended after the volunteer helps. Another motivation is ‘Impulse’ where volunteers listen to their inner voice and move because of an urge from their heart – their feelings to get involved in helping. As expressed by volunteers, ‘Impulse for others’ (RESP-02, 45 years old, female) and, ‘Help, relieve and make us smile happily’ (RESP-08, 40 years old, female).

#### Theme 4: Attitudes that need to be possessed when helping

This theme, as expressed by a participant ‘Empathise’, (RESP-07, 40 years old, female) and another respondent, ‘Empathy to fellow human beings’ (RESP-45, 51 years old, female). The meaning of helping is a form of empathy, and there is sincerity of volunteers to the distress of others. ‘Giving help wholeheartedly with no strings attached’ (RESP-34, 25 years old, female). Empathy means being able to understand and comprehend the situation of the other party, and with a sincere attitude then shape helping behaviour into meaning.

#### Theme 5: Cooperation with other parties

Participants responded to the meaning of helping as a behaviour that is realised from the interaction of two or more parties, namely between the volunteer who helps and the other party, at least the party being helped, including with other parties, fellow helpers or volunteers. There is an element of cooperation required in this meaning of helping because it involves several parties. ‘Giving something to those in need can be moral and material to ease the burden on a person or group’ (RESP-49, 52 years old, female) is a participant’s response that shows the need for cooperation because it is not done alone.

### Study 2

Study 2 on psychosocial meaning produced seven themes identified as psychosocial meaning as shown in Table 5. The sub-theme ‘Housing and infrastructure’ and the sub-themes ‘Treatment’ and ‘Groceries’ indicate the types of assistance that are ‘Material’ in psychosocial support activities. The theme ‘Lighten the burden’ shows the purpose of the psychosocial support provided by volunteers, which can be ‘Dikaruhke’ or greeted, or ‘Prayed for’ and ‘Fulfilled basic needs’. The theme ‘Non-material forms’ shows the types of psychosocial support that are non-material and very diverse, which is illustrated by the sub-themes ‘Psychoeducation’, ‘Social Services’, ‘Play therapy’, ‘Art therapy’, ‘PFA’, ‘Counselling’, ‘Psychological and social support’, ‘Providing post-disaster comfort’, ‘Therapy or trauma healing’, ‘Recovering after loss’ and ‘Motivation’, all of which are understood by volunteers as forms of psychosocial support. On the other hand, the themes ‘Facing certain circumstances or objects’ and ‘Connecting with others’ show the situations faced by volunteers when providing psychosocial assistance or support. The theme ‘Recovery’ shows the expected conditions for those who are given psychosocial support. The theme ‘Help or support’ shows the nature of the activity of psychosocial support, namely in the form of efforts by outsiders to disaster survivors who are considered in need.

**TABLE 5:** Theme findings meaning of psychosocial support for volunteers.

No	Theme	Sub-theme
1	Material	Housing and infrastructure Treatment Groceries
2	Lighten the burden	Dikaruhke (greeted) Prayed for (spiritual) Fulfilled basic needs
3	Non-material forms	Psychoeducation Social Services Play therapy Art therapy PFA Counselling Psychological and social support Providing post-disaster comfort Therapy or trauma healing Recovering after loss Motivation
4	Facing certain circumstances or objects	Unexpected situation The situation is as expected
5	Connecting with others	Individual Family Group or community
6	Recovery	Empower Back to normal
7	Help or support	According to the needs Less appropriate or more than needed

No, number; PFA, Psychological First Aid.

#### Theme 1: Material

This theme summarises the types of psychosocial support that are material in nature, which is an equally important part of non-material psychosocial support. This finding reinforces the need for a balance of types of psychosocial support in psychosocial support programmes by paying attention to the basic needs of disaster survivors, namely Housing and infrastructure medical treatment and basic necessities. As expressed by a participant, ‘Assistance aimed at people who are in disaster. Assistance is tailored to the needs’ (RESP-4, 63 years old, female) and another participant said, ‘Present in the midst of the disaster survivors. And help both PFA (Psychological First Aid), providing basic necessities, treatment etc.’ (RESP-11, 23 years old, female). ‘Social assistance, for example, basic necessities’ (RESP-24, 33 years old, male).

#### Theme 2: Lighten the burden

This theme indicates the purpose of psychosocial support activities. Whatever is done should refer to this goal, not for other purposes, let alone add to the problems of disaster survivors. From just being greeted (Dikaruhke in Javanese idiom) to being prayed for and given the materials needed, both the process and direction must refer to the purpose of providing psychosocial support, which is to lighten the burden of disaster survivors. For example, the response of the participant from Palu, ‘Providing support [*or*] assistance, listening to them, counselling’ (RESP-07, 40 years old, female) and the respondent from Kediri, ‘Attention through greetings’ (RESP-57, 53 years old, male).

#### Theme 3: Non-material forms

This theme provides an overview of the types of non-material psychosocial support. Participants gave very diverse responses to this theme, ranging from simply listening activities, providing a sense of security after the process of losing property or family, to providing skills to specific psychological therapy activities such as art therapy. The participant from Kulon Progo mentioned, ‘Comforting, accompanying’ (RESP-61, 54 years old, female) as a type of psychosocial support, the participant mentioned, ‘Help with emotional problems, for example, conveying motivation in the life experienced’ (RESP-50, 43 years old, male) regarding providing motivation. Meanwhile, a participant mentioned psychosocial support, ‘providing encouragement to be able to survive and recover in mind and spirit for victims of a social event. Examples: entertainment, skills assistance, disaster risk understanding assistance’ (RESP-56, 48 years old, male).

#### Theme 4: Facing certain circumstances or objects

Psychosocial support also conditions volunteers to deal with situations that are often in accordance with the initial description at the time of preparation to help, but it is also not uncommon for volunteers to deal with situations beyond their expectations. The participant mentioned psychosocial support as, ‘Giving what is needed’ (RESP-10, 40 years old, male) so that it means that whatever is seen and faced as the needs of disaster survivors, it is necessary to help fulfil their needs in psychosocial support by volunteers. There are no conditions that are always the same according to the theory, so volunteers, when going to the field, are very important to be ready and open to face whatever the conditions are.

#### Theme 5: Connecting with others

This theme provides information emphasising that in psychosocial support, disaster survivors are subjects, not objects, and they are people. ‘Being present in the environment of disaster survivors. And helping both PFA (Psychological First Aid), providing basic necessities, treatment, etc.’ (RESP-12, 23 years old, female). Being present gives meaning to what is important, not just running errands and being done with it. This reminds the importance of a well-built relationship, humanising the disaster survivors, instead of making them the target of a programme that is passive, let alone creating dependence and negating the principle of independence in disaster survivors.

#### Theme 6: Recovery

A participant from Palu mentioned, ‘Support to help restore a person’s psychological and social state in dealing with the circumstances they face such as the loss of property or loved ones’ (RESP-01, 33 years old, female). Restoring means restoring the person to their original condition, rather than making them dependent on the helper:

‘Psychological recovery assistance for people due to an event so that their condition can return to what it was before the event (e.g., psychological assistance for victims of natural disasters).’ (RESP-28, 45 years old, male)

#### Theme 7: Help or support

This theme shows the fact that the honesty of volunteers when helping is often not necessarily in accordance with the needs of the disaster survivors being helped. This awareness creates a strong urge to continue learning so that what is done when helping is really in accordance with what is needed, effectively achieves the purpose of helping behaviour and has a positive impact on the interests of those being helped. A female participant (RESP-4, 64 years old) said:

‘The help that is given to the people who are in distress … is tailored to the needs. Assistance is tailored to the needs. For example, helping children who are accepting of the disaster experienced (earthquake). Until a sense of comfort is achieved in the children being assisted.’

Likewise, a participant expressed her opinion:

‘Providing psychosocial support with the aim of restoring individuals, families or groups, after certain events (natural disasters and social disasters) so that they become strong individually or collectively can function optimally, have resilience in dealing with problems and become empowered and productive in living their lives. Examples: PFA (Psychological First Aid), psychosocial services, psychoeducational counselling services.’ (RESP-69, 64 years old, female)

The data also show that in participants’ responses to the question on the meaning of psychosocial, there appears to be a wide disparity, with participants’ responses showing a general to very specific understanding of volunteering. There were some respondents who wrote ‘don’t know’ or even wrote ‘don’t know many people’ which seems to be misunderstood as an antisocial term. The findings from the integration of studies 1 and 2 can be seen in Table 6.

**TABLE 6:** Matrix of study theme findings 1 and 2.

Theme	Study 2	
	Meaning of help	Meaning of psychosocial support
Study 1	**Values held by volunteers:** caring, meaningfulness of life, obligation, awareness, acts of love, humanism	**Material:** Volunteers’ helping behaviour in providing psychosocial support also involves assistance in the form of materials
	**Purpose in helping:** relief, giving help	**Lighten the burden:** The helping behaviour of disaster volunteers in providing psychosocial support must aim to lighten the burden on those being helped
	**Motivation of helping behaviour:** Making yourself happy, impulse	**Non-material forms:** The helping behaviour of disaster volunteers in providing psychosocial support is closely related to non-material things, mentioned in various activities
	**Attitudes that need to be possessed when helping:** sincerity, willingness, open to continue learning	**Facing certain circumstances or objects:** The behaviour of disaster volunteers in helping needs to be done with awareness of the importance of being sensitive to the existing situation
	**Cooperation with other parties:** Connecting with others	**Connecting with others:** Disaster volunteers in helping treat disaster survivors as subjects, not objects
		**Recovery:** Disaster volunteers focus on the goal of their helping behaviour, namely restoring the condition of disaster survivors
		**Assistance or support:** The principle of psychosocial support is assistance or support so its nature needs to be measured and limited by timely and realistic
	**Special themes or other findings study 1** **Volunteer Motto:** – Come-do-go home-Forget – Menang Cacak and Kalah Cacak (In Javanese idiom) – Family is something that must not be defeated even in the name of helping – Don’t get carried away by posting counterproductive social media statuses	**Special themes or other findings study 2** There is a very diverse understanding of psychosocial support; there is no understanding of the meaning of psychosocial support for natural hazard volunteer respondents in providing psychosocial support

Qualitative themes of psychosocial meaning synthesised in this study include: (1) Material: The helping behaviour of volunteers in providing psychosocial support also involves material assistance; (2) Lighten the burden: The helping behaviour of disaster volunteers in providing psychosocial support must aim to lighten the burden on the party being helped, not to cause conflict or other social disasters; (3) Non-material forms: The helping behaviour of disaster volunteers in providing psychosocial support is closely related to non-material matters, mentioned in various activities. So, the psychological and sociological-cultural elements are very closely related to the entire process of providing psychosocial support. What is given and how to assist must include paying attention to the culture and values of the local community; (4) Facing certain circumstances or objects: The behaviour of disaster volunteers in helping needs to be done with awareness of the importance of being sensitive to the existing situation, not imposing their own context but instead adjusting the context of the community being helped; (5) Connecting with others: Disaster volunteers in helping treat disaster survivors as subjects, not objects so that they are obliged to treat them well; (6) Recovery: disaster volunteers focus on the purpose of their helping behaviour, namely the restoration of the condition of disaster survivors to become empowered again, independent; and (7) Help or support: The principle of psychosocial support is assistance or support so that its nature needs to be measured and limited by time and realistic about the limitations of the helper.

## Discussion

This study identifies qualitative themes that are specifically perceived as helping behaviour of natural hazard volunteers in providing psychosocial support from the understanding of the meaning of helping and the meaning of psychosocial support according to participants. Data collection was carried out when the pandemic was still ongoing, although it was quite under the control of the government with the easing of mobility, so it was carried out online. Through these two qualitative studies, this research underlines that the behaviour of disaster volunteers is influenced by the understanding of the importance of helping and the meaning of psychosocial support.

This research shows that helping behaviour for volunteers is loaded with philosophical values. Volunteer mottos such as ‘Come-Do it-Go-Forget it’, ‘Menang Cacak and Kalah Cacak (In Javanese idiom)’, ‘Stay focused on family if needed’ and the principle of ‘Not uploading on social media what is done’, are things that refer to the keywords, namely sincerity and being a balance. This finding is in line with the theory proposed by Baron and Byrne (2005) that helping behaviour is an action that provides direct benefits to other individuals, without providing benefits to individuals who provide help. In addition, these findings are also in line with the theory of Kassin et al. (2014) regarding three aspects of helping behaviour, namely reward, empathy and altruism and egoism:

- Reward is the reward that individuals receive when they help. One of the reasons individuals help is to obtain psychological and material rewards. Individuals often feel happy when helping other individuals. The feeling of being a useful and meaningful person for others is a reward that volunteers get when helping. According to Putnam (2000), volunteerism is usually understood as social activities that improve social welfare, empower local communities and provide services that were previously absent or limited in that location. Participants find meaning in helping.
- Empathy is the feeling of understanding an individual’s perspective either directly or indirectly and feeling sympathy or compassion from that individual. Empathy has two important components, namely cognitive and emotional. The cognitive component of empathy helps individuals in perspective-taking so that individuals are expected to be able to perceive something according to their imaginative abilities. The emotional component is empathic care in the form of feelings of affection, sympathy and tenderness. The existence of concern in volunteers is the beginning of the birth of empathy.
- Altruism and egoism are things that cause individuals to perform helping behaviour. Individuals who help based on altruism have a desire to improve the welfare of other individuals and do not think about the rewards they will receive. Thus, the value of sincerity that dominantly appears in the motto conveyed by volunteers is a form of altruism. The results also show the findings of important values for the sustainability of helping behaviour in the midst of difficult challenges or situations. Although there are problems with finances and limited facilities when volunteers have the will, the limited facilities will make them creative and innovative. Willpower is also not just reckless but measurable. Volunteering does require a lot of time, so it cannot be done by people who work full-time. Volunteers who are ‘willing or strongly committed’ are usually not full-time workers. Individuals can also engage in helping behaviour due to egoism, which is the desire to improve their own well-being. Individuals perform helping behaviour to increase positive feelings within themselves. The existence of a sense of self-spirituality in volunteers is close to this concept. Volunteers feel positive emotions, such as pleasure, when they help.

This study also provides an overview of how the meaning of helping is understood similarly by volunteers, which aims to lighten the burden of others and provide assistance as a form of concern for the burden of others. This is in accordance with research conducted by Hogg and Vaughan (2002) and Baron and Byrne (2005), which state that helping behaviour is an act that provides more benefits for others who are helped, although there are benefits for the perpetrator who helps. The benefit for the helping actor, namely the volunteer, is to be able to smile happily when witnessing the party being helped and can be happy in accordance with the purpose of the help. This finding also explains that individual empathy has an effect on helping behaviour. Mazraeh et al. (2023) explained that helping behaviour can also provide encouragement to continue to do good in an environment affected by problems, which is driven by empathy and social skills. Krol and Bartz (2022) also explained that although the tendency of individuals to help is driven by their empathy, it does not explain enough. Recent research findings explain the role of a strong self-concept in understanding the situation, which will play a role in moving individuals to help. Perrykkad et al. (2023) also explained that the role of self-concept can make it easy for individuals to be more active in acting; this is due to the individual’s beliefs or self-efficacy.

In addition to this, an interesting finding is that helping can also be interpreted as intrinsic. In Cryder, Loewenstein and Seltman’s (2013) research, helping behaviour is not only related to kindness but it also provides personal satisfaction in solving existing problems. Individuals in helping behaviour can be moved by personal goals or achieve their own satisfaction. Dury et al. (2023) explain that helping behaviour can also have the effect of helping again. This also happens during a pandemic or bad situation. Ultimately, the effects of helping can make individuals feel better psychologically (Jia, Zhong & Xie 2021). Fundamentally, helping behaviour can be influenced by an individual’s personality, giving rise to functional motives in driving individuals to act (Mowen & Sujan 2005).

The findings of this study also show the keyword ‘burden’ that helping needs to be done according to the burden of the person being helped and the capacity of the helper, even though in reality it often does not match the needs of the person being helped. As stated by Schein (2009), helping behaviour is complex; it can be beneficial to the person being helped, but it can also be unhelpful.

In the context of disasters, individuals will be more easily moved to help after seeing the situation that occurs especially when reciprocal altruism occurs (Silva et al. 2009). When one individual provides help to another, it can have an impact in the future and disaster situations; these emotions often arise to provide encouragement to help. Helping behaviour in disaster volunteers refers to actions taken by individuals to help others during and after a disaster. This can range from providing direct physical assistance, such as rescue operations, to offering emotional and psychological support. Volunteers can be either formally trained individuals or untrained spontaneous helpers who respond to the needs of disaster survivors (Carvajal-Aguirre et al. 2017; Tang et al. 2024; Silva et al. 2009).

Thus, from this research, it is illustrated that helping behaviour is a form of concern to relieve people, which is done sincerely. For volunteers, helping is to make their lives more useful and meaningful for others, even though sometimes the help given is not in accordance with the needs of the person.

While the interesting thing about the meaning of psychosocial is that some respondents interpret psychosocial as non-material assistance, there are also those who interpret it as a whole or series of assistance provided for the purpose of support to disaster victims, both material and non-material, so that they are able to return to their ‘normal’ position. This is in accordance with the psychosocial definition of Smet (1994) which states that there are four dimensions of social support: (1) Emotional support: in the form of expressions of feelings of care, empathy, and concern for other individuals; (2) Appreciation support: in the form of positive expressions as a form of respect for other individuals; (3) Instrumental support: in the form of direct assistance in the form of material; and (4) Informative support: in the form of providing advice, suggestions or feedback to individuals. The findings of this study reinforce the importance of the entire process of providing support or assistance to beneficiaries; that psychosocial support is not only limited to mental support in the form of counselling or play therapy for example, but also how physical and material assistance is channelled in the right way. That is the real meaning of psychosocial support, restoring disaster survivors in a way that is humane, independent, prosperous (physically and mentally) and empowering (Koentjoro & Andayani 2007).

Another finding was that some respondents did not know the meaning of psychosocial support, and some respondents mentioned specific examples of psychosocial support. This indicates a gap in the level of understanding among disaster volunteers, which can impact the process of providing psychosocial support and the targeted outputs and outcomes. This is in line with the advice for disaster intervention that the general public who want to help in handling natural hazards should be equipped with sufficient knowledge and skills to help victims so that officials and volunteers from organisations do not feel bothered by their presence (Utomo & Minza 2018). In terms of experience in the post-disaster psychosocial support process, respondents stated that this social aspect of assistance is very important and needed, with keywords that often appear are assistance, disaster, social, psychological, mental and support. The second study also showed that simply being cared for, visited, asked for news and listened to are forms of psychosocial support. This is in accordance with one of the dimensions of psychosocial support from Smet (1994), namely emotional support.

## Conclusion

Based on the research findings that have been described and discussed in the previous section, it can be concluded that the helping behaviour of natural hazard volunteers in providing psychosocial support is influenced by the meaning of helping and the meaning of psychosocial support. Helping is interpreted as helping to lighten the burden on others as a form of concern for others, to make life more meaningful and get the satisfaction of spirituality. Then, important values in helping behaviour are sincerity, willingness, openness to learn, willingness to sacrifice and flexibility. In general, psychosocial support means a form of assistance provided to post-disaster survivors to improve the social psychological condition of individuals/communities. However, as noted, the research findings show that there is a very diverse understanding of psychosocial support, with a large variance. There is no common understanding of the meaning of psychosocial support for volunteer respondents. Volunteers’ helping behaviour in providing post-disaster psychosocial support is any form of assistance provided during the post-disaster period, to improve social psychological conditions so that the beneficiaries’ burden becomes lighter. The forms of assistance provided, such as sharing stories, listening to complaints, accompanying, supporting, strengthening and other things that are non-physical/immaterial, as well as material assistance, which is a series of post-disaster volunteer helping behaviour packages.

Thus, helping behaviour as a form of psychosocial support is not only in the context of psychic assistance but also included in the entire series of assistance when the delivery of assistance is provided in a supportive manner, answering the needs of disaster survivors so that they can recover from adversity.

Understanding the personal meaning of helping, which is full of humanistic and spiritual values for volunteers, shows the need for every decision-maker to reward and treat volunteers with humanistic and spiritualistic approaches according to their local values. A materialistic approach is not the only appropriate way to support the sustainability of volunteers’ helping behaviour. Continuous efforts are also needed to preserve local wisdom values in disaster risk reduction programmes, especially related to volunteer helping behaviour. The understanding and expectation that psychosocial support is not only limited to non-physical and mental assistance but also includes how the logistical assistance process is provided, encourages the importance of volunteer helping behaviour to be managed synergistically and holistically for both logistical and non-logistical volunteers.

In addition, it is important to conduct continuous training and education efforts to support the understanding of the meaning of psychosocial support for volunteers so that the direction and energy of volunteers in helping behaviour will be more effective in reducing the negative impacts of disasters. The limited capacity of volunteers in understanding psychosocial programmes can be improved in the form of training or similar activities that are measurable, monitored and sustainable. Suggestions for future researchers are very open to research topics related to local wisdom values for strengthening the capacity of post-disaster volunteers.

The practical and specific implications of the research findings are as follows:

- For policymakers in disaster governance: National Disaster Management Agency or BNPB, Regional Disaster Management Agency or BPBD, Central Government and Regional Government at the provincial level to the village level. It is necessary to mainstream psychosocial support for disaster volunteers as an important issue in disaster programmes, not just an additional programme in disaster governance. Budgeting, needs assessment and interventions need to be aligned with the needs of the local wisdom of the volunteer community itself, not top-down but bottom-up from the perspective of disaster volunteers. This is to support the sustainability of the volunteer community so that it does not depend on assistance from outside the community.
- For government and civil society organisations: It is necessary to design and develop programmes that support the capacity building of volunteers that increase the resilience of successful post-disaster psychosocial interventions. In particular, material on mapping the potential of local wisdom psychosocial support and how to build and preserve it in line with the presence of Western psychosocial support approaches. The intersection of local wisdom with a scientific approach will go a long way to bridging community sustainability.
- For future researchers: In line with the findings of Study 1, psychosocial support among volunteers should be further researched to identify the local wisdom of volunteers so as to find resilience strategies of the volunteer community that are suitable for the needs of indigenous peoples and local communities.
